# Understanding Regulation of Metabolism through Feasibility Analysis

**DOI:** 10.1371/journal.pone.0039396

**Published:** 2012-07-09

**Authors:** Emrah Nikerel, Jan Berkhout, Fengyuan Hu, Bas Teusink, Marcel J. T. Reinders, Dick de Ridder

**Affiliations:** 1 The Delft Bioinformatics Lab, Department of Intelligent Systems, Delft University of Technology, Delft, The Netherlands; 2 Systems Bioinformatics IBIVU, Faculty of Earth and Life Sciences, Vrije Universiteit, Amsterdam, The Netherlands; 3 Kluyver Centre for Genomics of Industrial Fermentation, Delft, The Netherlands; 4 Netherlands Consortium for Systems Biology (NCSB), Amsterdam, The Netherlands; 5 Netherlands Institute Systems Biology (NISB), Amsterdam, The Netherlands; Hospital for Sick Children, Canada

## Abstract

Understanding cellular regulation of metabolism is a major challenge in systems biology. Thus far, the main assumption was that enzyme levels are key regulators in metabolic networks. However, regulation analysis recently showed that metabolism is rarely controlled via enzyme levels only, but through non-obvious combinations of hierarchical (gene and enzyme levels) and metabolic regulation (mass action and allosteric interaction). Quantitative analyses relating changes in metabolic fluxes to changes in transcript or protein levels have revealed a remarkable lack of understanding of the regulation of these networks. We study metabolic regulation via feasibility analysis (FA). Inspired by the constraint-based approach of Flux Balance Analysis, FA incorporates a model describing kinetic interactions between molecules. We enlarge the portfolio of objectives for the cell by defining three main physiologically relevant objectives for the cell: *function*, *robustness* and *temporal responsiveness*. We postulate that the cell assumes one or a combination of these objectives and search for enzyme levels necessary to achieve this. We call the subspace of feasible enzyme levels the feasible enzyme space. Once this space is constructed, we can study how different objectives may (if possible) be combined, or evaluate the conditions at which the cells are faced with a trade-off among those. We apply FA to the experimental scenario of long-term carbon limited chemostat cultivation of yeast cells, studying how metabolism evolves optimally. Cells employ a mixed strategy composed of increasing enzyme levels for glucose uptake and hexokinase and decreasing levels of the remaining enzymes. This trade-off renders the cells specialized in this low-carbon flux state to compete for the available glucose and get rid of over-overcapacity. Overall, we show that FA is a powerful tool for systems biologists to study regulation of metabolism, interpret experimental data and evaluate hypotheses.

## Introduction

In their natural habitat, most microbes are exposed to constantly changing physical and chemical environments. To perform optimally in these conditions, they must finely regulate their metabolism. Understanding how microbes regulate metabolism to achieve a desired objective, or how they adapt to changing conditions, is a major challenge [1]. Quantitative analyses relating changes in metabolic fluxes to changes in transcript or protein levels have further revealed a remarkable lack of understanding of the regulation of these networks; it remains unclear how and to what extent metabolic networks are regulated through the modulation of enzyme levels [2]. Regulation analysis has shown that metabolic networks are controlled via non-obvious combinations of metabolic and hierarchical regulation [3], [4].

Thus far, in systems biology two main model-based approaches are used to study metabolic regulation: *top-down* and *bottom-up*. The top-down approach employs genome-wide constraint-based modeling techniques, such as Flux Balance Analysis (FBA), to find viable intracellular flux distributions based on measured external fluxes and thermodynamical considerations. Constraint-based models have been shown useful in exploring cellular capabilities of biological systems and have enabled in silico characterization of several phenotypic features, such as growth yield under gene knockouts (see [5] for a review). However, an inherent limitation of constraint-based models is that they are based solely on stoichiometry and thus are limited to predicting steady-state flux distributions. In general, they do not contain explicit regulation terms and cannot predict the effect of gene or enzyme dosage via knock-ins or point mutations. It is however possible to constrain the solution space by incorporating series of physiological parameters [6] or additional -omics data [7], or by assuming certain objectives for the cell [8]. The list of such objectives ranges from maximization of biomass to minimization of redox potential. A systematic evaluation [9] revealed that *Escherichia coli* employs different objectives under different conditions.

In contrast, the bottom-up approach combines detailed kinetic models with the theorems of Metabolic Control Analysis (MCA, [10]) or Biochemical Systems Theory (BST, [11]) to study regulation. While kinetic models describe metabolic reaction rates as a function of enzyme levels and metabolite concentrations, the inverse models describing (changes in) enzyme levels required to obtain desired metabolite concentrations or reaction rates are more useful for studying regulation. However, as most kinetic models are highly nonlinear, explicit inversion is often impossible. Both within the framework of MCA and BST, a number of approximative kinetic formats [12]–[14] have therefore been proposed as a solution [15]–[17]. Although useful, these kinetic descriptions usually offer limited mechanistic insights.

In this paper, we employ a method of studying regulation, Feasibility Analysis (FA), combining elements of bottom-up and top-down approaches. FA starts from an explicit kinetic model describing the interactions between enzymes and metabolites. Inspired by the well-established constraint based approach of FBA, it then defines a number of physicochemical constraints on the cell, as well as three physiologically relevant objectives: function, robustness and temporal responsiveness, for which quantitative measures are introduced. Assuming that the cell follows one or a combination of these objectives, FA then searches for (a) set(s) of enzyme levels necessary to achieve these. Given the problem of inversion of general non-linear kinetic models, FA uses a straightforward sampling-based method, commonly used for various computational biology purposes, e.g. for ensemble modeling [18], or modeling the uncertainty in biochemical reaction networks [19], [20]. For each sampled set of enzyme levels, the kinetic model is integrated to steady state and objective measures are calculated on the resulting phenotype. We call the subspace encompassing all feasible enzyme levels the feasible enzyme space. Once this space is constructed, we can study how different objectives can (if possible) be combined, or evaluate the conditions under which these objectives are traded-off.

A similar approach of using physiological constraints to find feasible sets of enzyme levels was successfully applied to identify the required changes in gene expression in yeast upon heat shock [21] and, more generally, to attain certain cellular adaptive responses [22]. This method was adapted to study general design principles of metabolic networks, employing optimization techniques to explore the space of feasible enzyme levels [21], [23]. While mathematically advanced, it is derived from a specific type of approximative kinetic model (Generalized Mass Action or GMA models), which limits its general use. FA aims (1) to generalize the GMA-based analysis by defining more generic, quantitative objectives that can be evaluated for any kinetic model; and (2) to get deeper understanding of regulation by explicitly incorporating the modes of regulation (metabolic or hierarchical) under physiological constraints and objectives.

The feasible enzyme spaces found by FA can also be used to enhance currently available kinetic models. These models are usually derived starting from an *ab initio* selected set of kinetic interactions; subsequently, parameter values are set or estimated by fitting to a (small) number of measurements. Methods to expand/shrink the model by adding/removing interactions and inspect the feasibility of the resulting models are of great interest. Using FA, we can thus discriminate between available hypotheses on how metabolism is regulated and evaluate potential changes in model structure.

In this paper, we first describe FA in detail, listing a number of constraints and introducing quantitative measures for the proposed objectives. We then exemplify the approach using two cases: (1) an illustrative small model with tractable kinetics and (2) a larger dynamic model of yeast glycolysis [24]. For yeast glycolysis, we analyze two scenarios: the adaptation of yeast cells during long-term chemostat cultivation under carbon limitation and the regulation of hexokinase to infer robustness to the glycolytic pathway. In each case, we also perform regulation analysis to determine the modes of regulation, and inspect on the relation between the physiological objectives and hierarchical or metabolic regulation. Additionally, we employ FA to investigate putative regulatory links, by extending the corresponding metabolic model with novel interactions and studying the changes obtained in the feasible enzyme space. We end with a discussion of our results and an outlook on further applications and possible extensions of feasibility-based approaches in systems biology.

## Results and Discussion

Biological systems constantly adapt to their environment and regulate their metabolism for optimal performance. In this paper, we study this regulation at a system level and use *feasibility analysis* (FA), considering physiological constraints and a list of potential objectives. We first describe these constraints and objectives and then apply FA to analyze two illustrative cases, a toy model and a model describing the glycolysis in yeast.

### Feasibility Analysis

**Figure 1:**
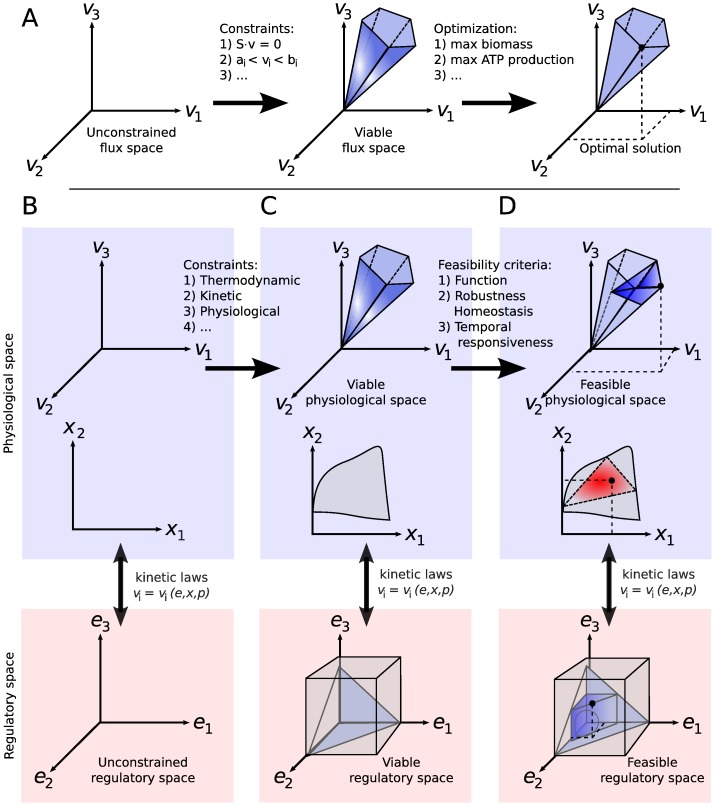
Feasibility Analysis (FA) explained. Panel fig:Feasibility-FBA illustrates constraint-based modeling often used within Flux Balance Analysis, starting from the unconstrained solution space and ending in the optimal solution (adapted from [8]). Feasibility Analysis is inspired from this constraint-based approach and combines it with the molecular rigor of a detailed kinetic model. The regulatory and physiological spaces are connected to each other with available kinetic rate equations for each reaction (usually a non-linear function of enzyme levels 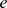, metabolite levels 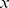 and kinetic parameter set 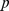). Under a number of constraints (e.g. thermodynamic, kinetic etc), only a subspace of both the enzyme and physiological space in panel fig:Feasibility-Spaces is viable, i.e. fulfills the constraints, as represented in panel fig:Feasibility-Allowed. Considering the list of feasibility criteria, only a subspace of this viable space is also feasible (panel fig:Feasibility-Feasible). The feasible enzyme space is constructed by evaluating the list of feasibility criteria for each physiological state in the viable space. The final feasible enzyme space can further be inspected within the scope of regulation analysis.

Figure 1 illustrates our overall approach. FA is inspired by the constraint-based approach used in FBA where an initial flux space is delimited by thermodynamic, mass balance and capacity constraints and the model is then optimized for a certain predefined objective to find the operational point or subspace (panel fig:Feasibility-FBA). Central to our FA approach, we incorporate a detailed kinetic model, taking mechanistic interactions between the enzymes, metabolites and rates quantitatively into account. The multi-dimensional space composed by enzyme levels 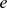, which we call enzyme space, is mapped to the physiological space (containing fluxes 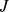 and metabolites 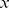) by the parametrized kinetic model.

We start by considering a large range of enzyme levels as the initial enzyme space. To construct the *feasible enzyme space*, this set should further be constrained. However, direct measures that can be applied as constraints are generally available for the physiological space only. In theory, since the enzyme space is mapped to the physiological space with the kinetic model, constraints in one space can be translated into the other by simply inverting the kinetic model. Yet, this inversion is generally not possible in practice due to the non-linear nature of the system. To solve this, similar to [19], we use Monte Carlo (MC) sampling. For each MC sample (a point in the enzyme space) the kinetic model is simulated until it reaches a steady state, yielding the corresponding point in the physiological space.

We first apply the “hard constraints” (thermodynamic, mass balance etc) on the physiological space and, via the kinetic model, on the enzyme space. These constraints yield the viable enzyme and physiological space. Next, we evaluate each feasibility criterion for each of the viable physiological states. The labels “feasible” or “infeasible” are thus assigned to each state and the feasible space is constructed (panel fig:Feasibility-Feasible). Finally, hierarchical regulation analysis can be applied to inspect where metabolism is regulated mainly hierarchically or metabolically, allowing to study the relation between physiological objectives and type of regulation (see Methods for more details).

### Constraints

The first step in FA is the application of the hard constraints. We take *thermodynamic*, *stability*, *kinetic*, *capacity* and *total protein* constraints into account (See Methods for a more formal definition of these constraints). We start by demanding that every biochemical reaction should obey thermodynamic laws. In general, this is formulated as discrete irreversibility constraints for fluxes through reactions operating far from equilibrium. When measurements on metabolites are available, thermodynamic properties such as Gibbs free energy can be calculated [25], which can be further used as continous constraints. Next, we consider stability, requiring that for each sampled point in enzyme space, the resulting model should be stable. As an approximation, this can *a priori* be computed by calculating the eigenvalues of the jacobian of the system at a selected steady state and requiring that all should have negative real parts. Then, owing to the available kinetic model, we take kinetic constraints into account. The relation between an enzyme, the metabolites and the rate for any reaction is constrained by its kinetic law. When extracellular fluxes are known and the entire network is considered, if any two of either enzyme, independent metabolite or intracellular flux levels are fixed, the third can be deduced using this set of laws for each enzyme in the network. Next, the capacity constraints provide upper limits for fluxes. Lastly, we assume that the cell economizes the change in total enzyme levels, so that when adapting to a new environment, the total enzyme level is kept within limited range. We also note that, though the total enzyme level is constrained, individual enzyme levels can vary independently within the allowed range.

### Objectives

FA continues by further constraining the viable space to obtain the feasible space, by considering a number of quantitiative, physiologically relevant objectives. Three feasibility objectives related to *function*, *robustness*, *homeostasis* and *temporal responsiveness* are proposed (for formal definitions of each of the criteria, see Methods).

#### Function

Biological systems have evolved to function optimally in a given environment. Within FBA, this optimal function is considered to be a flux towards a pathway, usually the growth rate; yet alternative optimality criteria such as minimization of uptake rate or redox potential provide adequate prediction of flux distribution [9]. Generalizing this, we consider a set of enzymes as functionally feasible if that set yields optimal (or near optimal) flux for a selected pathway. We also note that in FA, the total enzyme levels are constrained when maximizing flux, considering therefore the cells as optimal strategists for the use of resources, from a cost-benefit point of view [26], [27].

#### Robustness and Homeostasis

Robustness and homeostasis are two fundamental characteristics of biological systems [28, and references therein] and has long been recognized and studied from many aspects [29]–[33]. Following the definition in [28], we consider *robustness* as a property of systems that maintain their function under perturbations and uncertainty, and *homeostasis* as maintaining the state via coordinated physiological processes. Despite detailed qualitative descriptions [34] and *ad hoc* defined metrics, (e.g. [32], [35]), a general measure to quantify robustness in metabolism is lacking. To adress this, we first concretely define *state* and *function* for a given metabolic network as metabolite levels and the flux for a selected pathway in that network, respectively. We then consider the changes in enzyme levels as *perturbations*. To quantify robustness and homeostasis, we propose to use the metrics defined within the framework of MCA, namely co-response coefficients (see methods). Where MCA's control coefficients quantify relative change in one variable (state or function) upon change in another variable (perturbation), co-response coefficients measure the ratio of relative change in *two* different variables (state and function) in a network, resulting from a change in a third variable (perturbation). This coefficient is especially interesting to measure robustness and homeostasis of the network, since all three entities can be in different parts of the network.

We consider a set of enzyme levels to be feasible with respect to robustness if the function is maintained (or changes marginally) upon a change of level of any of the enzymes in this set. In that case, metabolite levels are expected to change, resulting in a small co-response coefficient for robustness (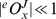). Similarly, we consider a set of enzyme levels to be homeostatically feasible, if the state is maintained (or changes marginally) upon a change of enzyme levels of any of this set, resulting in a small overall co-response coefficient for homeostasis (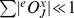, note the swap of indices for 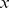 and 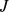) for a series of metabolites located on a pathway, taking into account the global coordination in the network.

#### Temporal responsiveness

Temporal responsiveness reflects how quickly the network responds to perturbations or external stimuli. It is based on the dynamic characteristics of (a subpart of) the system, such as the response time. From an evolutionary perspective, it is likely that certain pathways or cell types are selected based on their fast (or slow) response to changes in their environment. The key importance of dynamic properties for the cell to adapt to external stimuli has been exemplified for metabolic [36] and signaling networks [37], [38]. We consider a set of enzyme levels to be feasible with respect to temporal responsiveness, if it results in a small turn-over time for a metabolite of interest.

### Illustration on a small network

**Figure 2:**
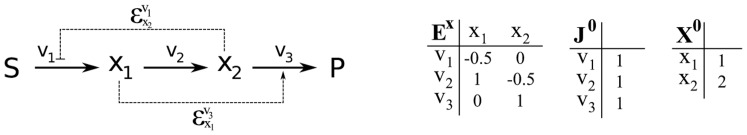
The small synthetic pathway used for illustration of the feasibility analysis. fig:ToyModel: The metabolic reaction network used. The solid arrows represent the base network and dashed lines indicate the additional kinetic interactions considered. fig:ElasticityMatrix: the reference steady state and the kinetic parameters for the small model.

Initially, to get insight in the shape and properties of the feasible enzyme space, we focused on a small model illustrated in Figure 2. We sampled 2

10^4^ enzyme level triplets (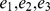), relative to their reference values, uniformly distributed in 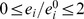. We then simulated the model to find the physiological space (the flux 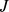 and metabolite levels 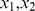 at steady state) corresponding to each triplet of enzyme levels. We then applied all constraints and finally evaluated each feasibility objective.

#### Constraints

**Figure 3:**
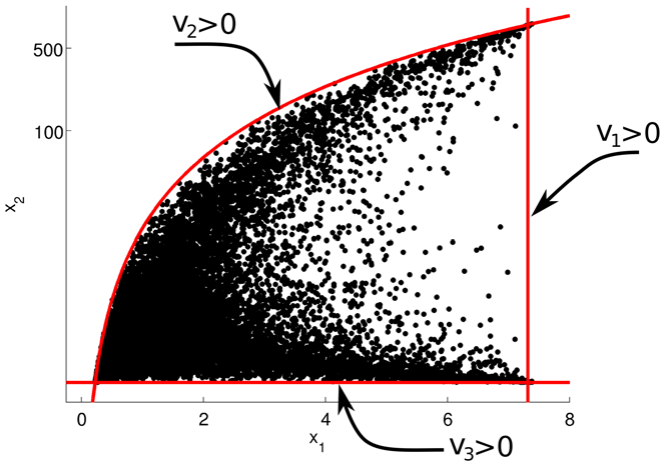
Thermodynamic constraints as limits to the physiological space. For the synthetic small problem, these constraints can be implemented before sampling. 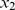 is presented in logarithmic scale.

For this small problem, the kinetic expressions allow to explicitly express metabolite levels as a function of enzyme levels. Starting by assuming linlog kinetics for each reaction yields:
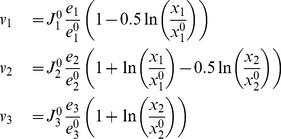
(1)Considering steady state mass balance, (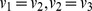) and substituting the values for 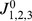 and rearranging yields:
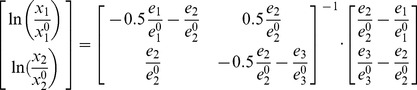
(2)where, 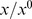 and 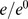 are the metabolite and enzyme levels relative to their reference state. Eq. 2 describes an explicit model (metabolite concentrations as functions of enzyme levels); fluxes can be obtained by substituting Eq. 2 into Eq. 1. To construct the feasible enzyme space, we start with the thermodynamic constraint and require the steady state flux and metabolite levels to be positive. The constraints on metabolites can analytically derived from Eq. 1, and are represented in Figure 3:
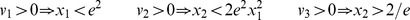
where 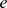 is the base of the natural logarithm. For the toy problem, all sampled enzyme level sets yielded physiological states that obey the thermodynamic and stability constraints. Finally, we constrain the total enzyme level to change by not more than 50% with respect to the reference state, noting that individual enzyme levels are allowed to vary freely within this constraint (i.e. 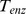 in Eq. 5 equals 0.5). By constraining the sum of all enzyme levels, around 85% of the sampled enzyme triplets remained viable.

#### Feasibility objectives

After applying the constraints, we analyzed the remaining viable space for each feasibility criterion. As a first step, we did not use any cut-off value (e.g. 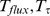) to discriminate a selected state as feasible or not; rather we visualized feasibility by assigning a color to each state according to a specific criterion (e.g. 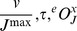; see Methods).

#### Function

**Figure 4:**
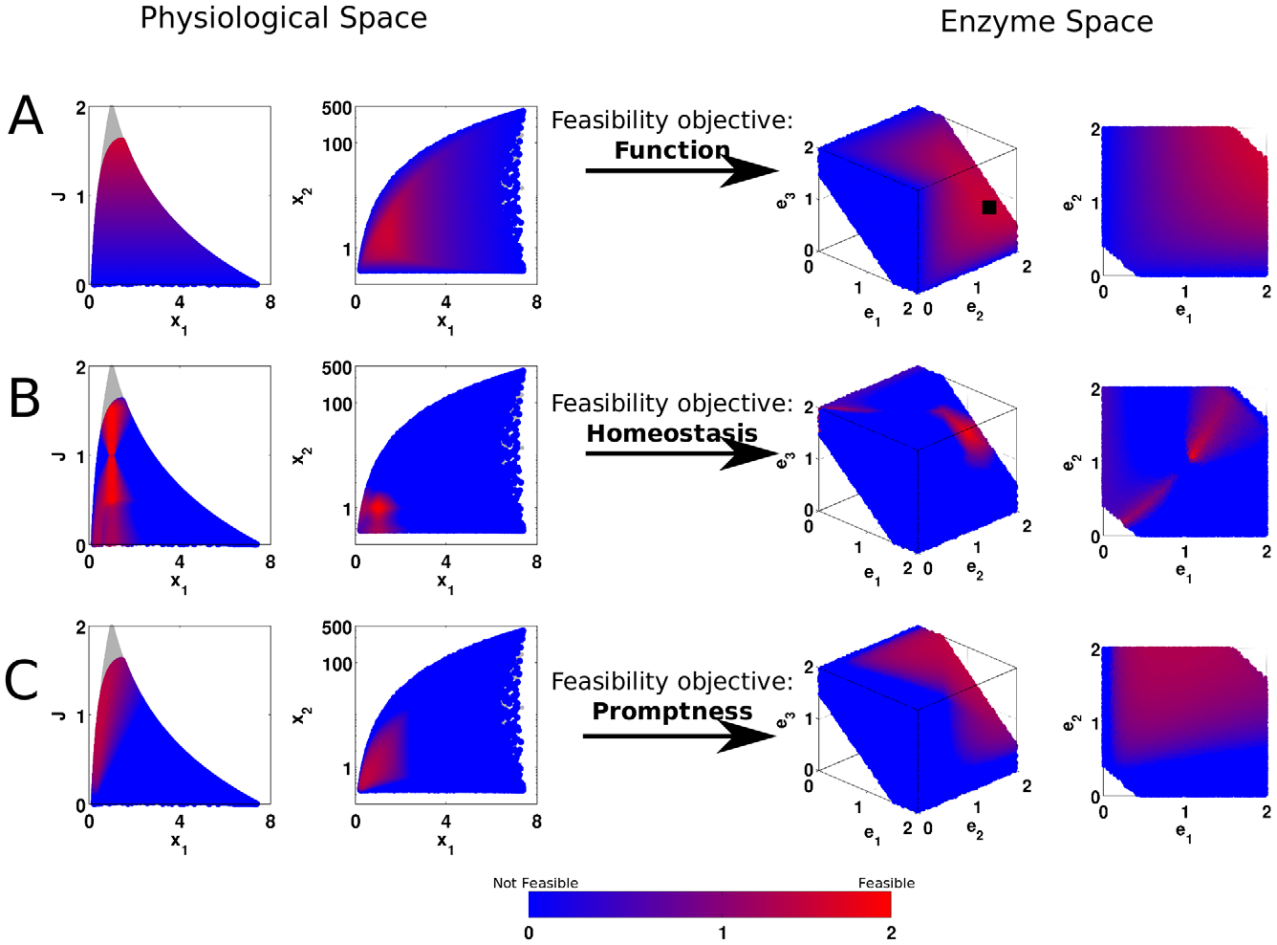
Feasibility analysis for the toy problem. The first two columns are the physiological space (first colum: flux vs. 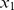, second column: 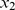 vs 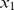), and the last two columns are the enzyme space (third column: viable enzyme space with all enzymes, fourth column: selected 2D slices from the third column at 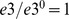). fig:FluxFeasToy: Optimal flux as feasibility criterion for function. fig:HomeostasisFeasToy: Homeostasis of the both metabolites as feasibility criterion. fig:PromptFeasToy: Turn-over time as feasibility criterion for temporal responsiveness. The red-blue color gradient indicates continuous values for the feasibility criteria in consideration, red indicates feasible states while blue indicates infeasible states The feasibility criteria are 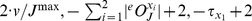, for function, homeostasis and temporal responsiveness respectively. The quantitative measures for homeostasis and temporal responsiveness have been changed sign and added offset for visualization purposes. The gray points in the physiological states are those for which the corresponding enzyme levels are outside the viable range, after applying the constraints. All axes in all plots are presented relative to their reference state, and 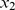 is presented in logarithmic scale.

We first consider function feasibility, by coloring each state according to the flux criterion defined in Eq. 6a. The resulting enzyme and physiological spaces are given in Figure 4(A). An immediate observation is that the flux increases as all three enzyme levels increase simultaneously (red points fall around the line 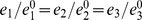, the main diagonal). The red colored points in the right plot show the enzyme levels that allow the network to achieve roughly the top 25% of possible fluxes. Note that FA takes the cost of the enzyme into account while evaluating the flux objective; for this problem, all enzymes are at equal cost as the optimum lies around the main diagonal. Since the total enzyme level is constrained, the flux is bounded and exhibits an optimal point (indicated by black square on the 3D plot). Furthermore, by taking metabolite levels into account, FA illustrates the effect of metabolic regulation. That is, in physiological space, metabolite level 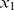 changes only over a limited range (increasing around 7-fold), while 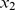 can increase up to 500 fold without affecting the flux, since 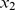 has no inhibitory effect on any of the rates.

#### Homeostasis and temporal responsiveness

Next, we analyze the homeostasis and temporal responsiveness feasibility. For homeostasis, the resulting enzyme and physiological space is presented in Figure 4(B), where the blue points in the right plot represent the enzyme levels that are homeostatically feasible, i.e. homeostasis can only be maintained if the enzymes in the network assume levels in the blue area of the enzyme space. Notably, to maintain homeostasis, all enzymes should change in concert, i.e. the blue points lie around the main diagonal (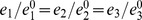) in the enzyme space. Given that metabolite levels change only marginally, while the flux levels do vary, the changes in flux are mainly attributed to changes in the enzyme levels. In order for the metabolite levels to remain unchanged while the flux is increasing, all enzyme levels should increase synchronously. Comparing Figures 4(A) and 4(B) we observe an interesting trade-off between homeostasis and function. Decreasing all enzymes simultaneously is homeostatically feasible, yet functionally not (the flux decreases). Similarly, increasing all enzymes simultaneously is homeostatically feasible, yet functionally not feasible (production of the enzymes would be too costly).

For temporal responsiveness (Figure 4(C)), we find that the effect of 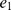 is small compared to that of 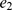 or 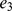: when either of these latter two is low enough, 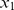 increases, therefore 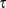 increases (red points). Similarly, a decrease in 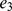 triggers the accumulation of 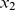, which in turn increases 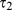 (not shown). This indicates that temporal responsiveness of this metabolic network is regulated by the last enzyme in this pathway, i.e. that the network has a “brake” at the end-point.

#### Combining feasibility objectives

**Figure 5:**
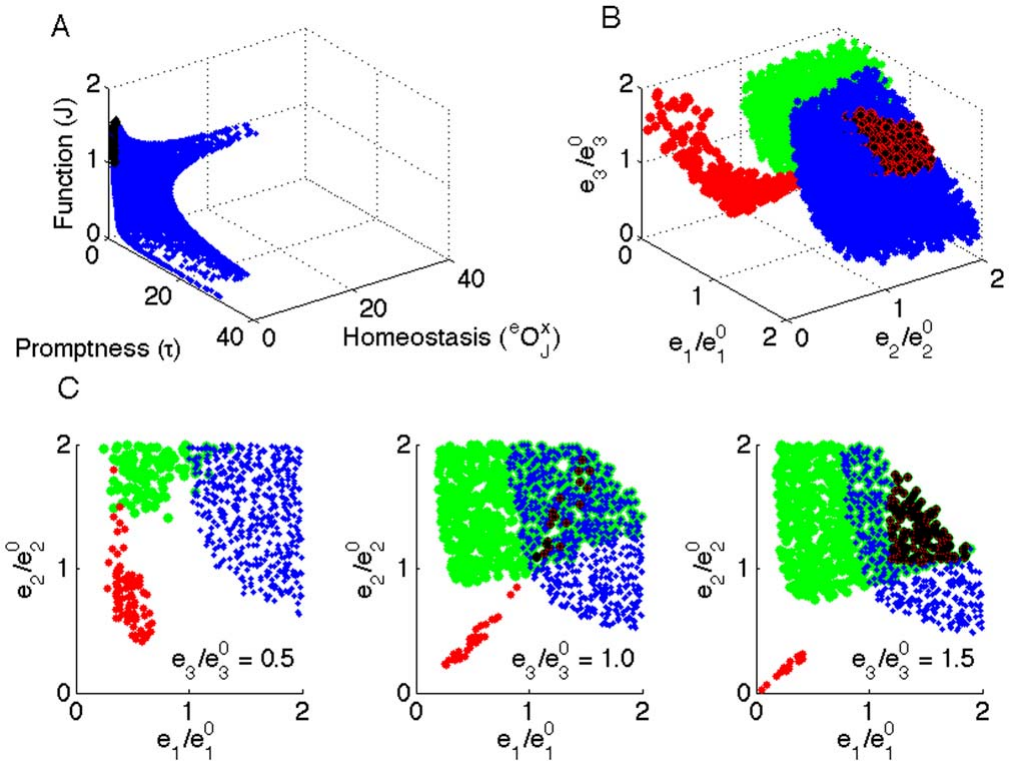
Combining feasibility criteria. fig:ObjSpace represents the objective space, where each feasibility criterion is taken along (no cutoff is used in this plot). fig:CombinedObj presents a 3D plot of the feasible enzyme space fig:CombinedObjLayers presents decompositions of the feasible enzyme space into a series of 2D slices, each differing by the value of 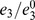 (indicated on the plot). Blue points describe the functionally feasible enzyme levels (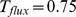 i.e. fluxes with top 25% are considered as feasible), red points are homeostatically feasible enzyme levels (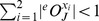), green points are the feasible enzyme levels considering the temporal responsiveness and black points are the states that are feasible for all three criteria.

We next investigated how the three objectives can be combined. For this, we first set a cut-off value for each criterion, as opposed to scanning the entire space as performed in the previous section. The results are given in Figure 5, showing the objective space (Figure 5(A)), the combined feasible enzyme space (Figure 5(B)) and a number of 2D-slices at different levels of 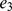 (Figure 5(C)). In the objective space, black points represent a very small subset of the feasible states satisfying all three objectives: high levels of 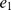, 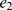 and 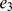. Interestingly, low levels of 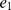, 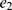 and 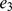 are homeostatically feasible, yet these enzyme levels result in a low flux, therefore functionally not feasible (Figure 5(B)). These states are especially interesting if a cell economizes on total enzyme levels. For the trade-offs, the optimal combination of objectives depends on the experimental context (see also the section “illustration on yeast glycolysis”). Another observation from Figure 5(C) is that only high levels of 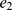, the enzyme that consumes 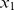, are feasible in terms of temporal responsiveness.

Next, we performed regulation analysis for this system and analyzed its relation with FA. To calculate the regulation coefficients (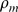 and 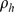) for each sampled point in the enzyme space, we considered the transition from the reference state to the perturbed state (sampled point) and made use of Eq. 7. We find that exclusively hierarchically controlled states (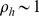) are homeostatically feasible. This is expected, since from the FA point of view, the homeostasis feasibility requires that the perturbation results in minimal changes in metabolite levels, and from the regulation analysis point of view the rate of a hierarchically regulated enzyme is exclusively affected by the level of that enzyme (therefore the metabolite levels do not change). Equivalently, exclusively metabolically controlled states (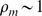) are also feasible with respect to robustness.

#### Regulation for feasibility: Feedback inhibition economically maintains homeostasis

In order to assess the effect of a given regulatory mechanism (e.g. end-product feedback inhibition), we modified the initial network and inspected the changes in the objectives and feasible enzyme space. We added a feedback inhibition of 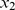 on 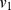, a regulatory mechanism ubiquitous in metabolic reaction networks (the dashed line from 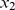 to 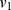 in Figure 2). We explored the model by changing the value of 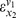 from zero to −0.5 (mild inhibition), up to −5 (strong inhibition).

**Figure 6:**
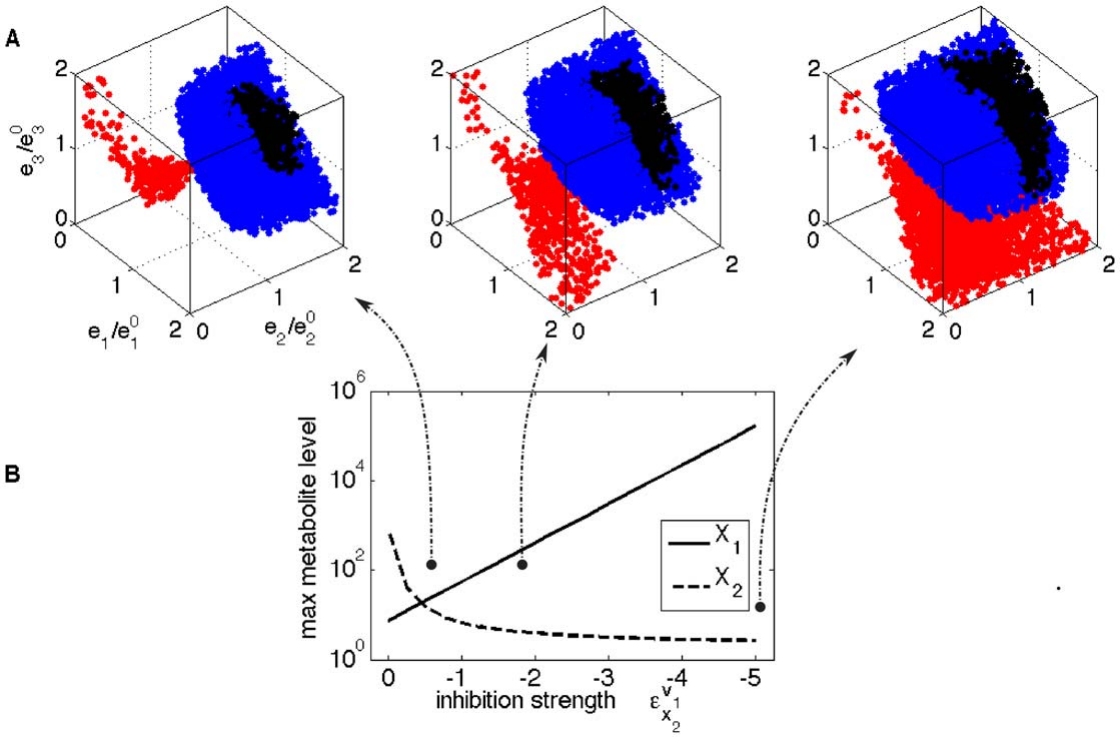
The effect of additional feedback inhibition of 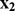
 **on** 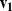 **on the feasible enzyme space with respect to homeostasis (** 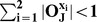 **) and function (** 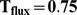 **).** fig:HomeostasisEv1x2: The feasible enzyme space for 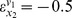 (left), 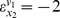 (center), and 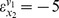 (right). In every subplot, red points: solely homeostatically feasible enzyme levels; blue points: solely functionally feasible enzyme levels; black points: feasible enzyme levels on both criteria. The axes for all 3 plots are the same, enzyme levels relative to the reference state. fig:AdditionalFeedbackEv1x2-MetLev: The maximum achievable metabolite levels as function of the inhibition strength.

The effect of this additional feedback inhibition on homeostasis feasibility is presented in Figure 6. It results in a decreased range of 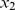, yet an increased range of 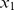 (Figure 6(B)). For the function feasibility, to have the same flux, higher 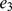 and lower 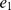 levels are needed with increasing feedback inhibition. For combining homeostasis and function feasibility, more states are feasible as inhibition strength increases (the feasible volume increases by 2.5 fold as 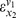 changes from 0 to −5, w.r.t. initial model). With increasing feedback strength, 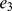 becomes more and more hierarchically regulated, in line with the previous result on combining regulation analysis with homeostasis feasibility. Similar observations, relating the effect of adding regulatory links in a metabolic network to the network sensitivity to perturbations, are reported in [39], [40]. The authors illustrated, using a frequency domain approach, that introducing feedback inhibition reduces the effect of perturbations on the output, but additionally showed that extreme feedback inhibition makes the system more sensitive to perturbations.

We also considered a possible feedforward activation of 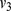 by 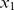 with various strengths (dashed line in Figure 2), and its effect on the feasible enzyme space. This activation further increases the control of 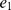 on the pathway flux (the flux control coefficients at the reference state are calculated as C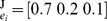 for 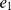, 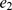 and 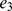 respectively at 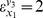.), as 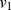 produces 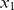 which in turn activates 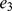. A further increase in 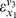 results in fewer homeostatically feasible states, illustrating that there is an optimal level of feedforward activation that maximizes the volume of homeostatically feasible space (data not shown).

### Illustration on glycolysis in yeast

Next, we applied FA to study yeast glycolysis under evolutionary pressure, especially focusing on trade-offs between alternative objectives. We used a model describing glycolysis in yeast [24] and analyzed two scenarios: the adaptation of the yeast cells during long-term chemostat cultivation under carbon limitation and the so-called “danger of turbo design”.

#### Feasibility analysis of prolonged chemostat cultivation of yeast

We first consider the scenario, where yeast cells were grown in a carbon limited chemostat for 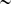
250 generations, resulting in a number of changes in their morphology as well as in metabolite and enzyme levels reported in [41]–[43]. Using FA, we explore the three objectives, find corresponding enzyme levels and compare these with the experimental measurements from [43].

In MC sampling the enzyme levels, we appended fermentor balances to the model in [24] and the extracellular metabolites were allowed to change freely. To construct the initial space, we randomly perturbed each enzyme in the network and monitored all resulting 17 fluxes and 13 metabolites. To obtain the viable enzyme space, each stable state was recorded and lastly all feasibility criteria were calculated for each state to construct the feasible enzyme and physiological spaces. We plotted the data from [43] on top of these spaces to inspect the actual changes in the enzyme levels.

**Figure 7:**
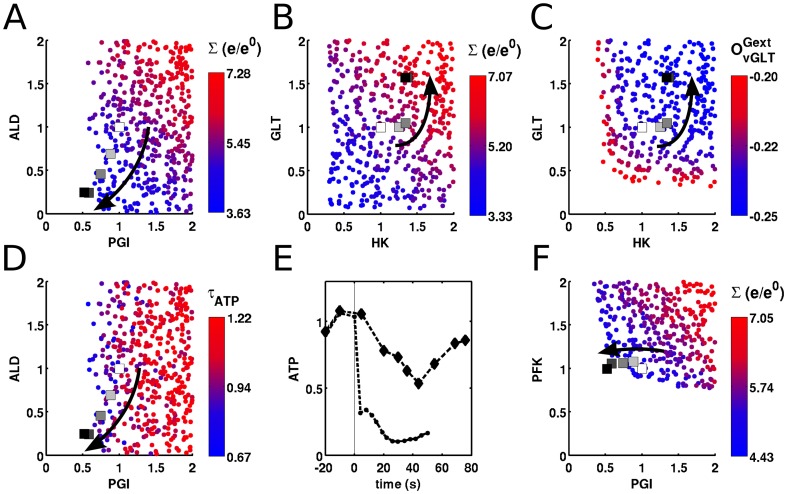
Feasibility analysis of the changes in enzyme levels during long term chemostat cultivation. In each plot, the dots (•) describe the sampled enzyme levels relative to reference state, colored according to the feasibility criteria specified in each plot above the color legend bar; the squares (▪) are the experimental data either from [41] or [43], white being the “wild-type” (10th generation) and black being the “evolved strain” (200th generation) and the arrow indicates the direction of the number of generations during the experiment (time). Enzymes not shown change only 10% from their reference state. fig:LongChemostatPGI-ALD: The function feasibility in terms of PGI and ALD, the color corresponds the total cost of the enzymes (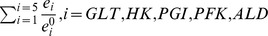). The experimental data from [43] shows that the cells evolved to an economized state.fig:LongChemostatGLT-HK: Function feasibility inspected for glucose transporter (GLT) and hexokinase (HK). The colouring is similar to fig:LongChemostatPGI-ALD, the sum of enzyme levels. The hypothesis on enzyme economy fails to predict the levels of these two enzymes for the evolved strain. fig:LongChemostatPGI-GLT: The evolution of glucose transporter and PGI enzymes inspected via homeostasis feasibility, as the co-response of extracellular glucose and uptake rate (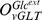). Cells evolve to a state where they are more apt to use extracellular resources. fig:LongChemostatPGI-ALD-ATP: The evolved state is predicted to allow yeast to respond quicker to external perturbations, as indicated by ATP temporal responsiveness feasibility as the color code for PGI and ALD. The experimental verification of this prediction is presented in fig:LongChemostatATPpulse where the response of ATP to a glucose perturbation (taken from [41]) is presented. The y-axis is the ATP level relative to the state before perturbation and x-axis represent the time in seconds. Evolved cells (•) respond quicker to glucose perturbation, when compared to wild-type cells (♦). fig:LongChemostatPGI-PFK: the function feasibility inspected for PFK and PGI. PFK levels, being already at the edge of the feasible space, can not further be decreased.

We first evaluated the hypothesis that the cells, under constant carbon influx, would economize the enzyme levels while coping with the constant carbon flux, as proposed in [41]. This hypothesis successfully predicts the enzyme levels for PGI and ALD (Figure 7(A), blue points towards to lower left corner having decreased cost). However, it fails to predict the change in enzyme level for the glucose transporter GLT and HK (Figure 7(B)). The levels of these two enzymes increase over the course of the experiment.

To explain this increase, we consider the homeostasis objective, and check the co-response coefficient of extracellular glucose and uptake flux for both enzymes (Figure 7(C)). To take the competitive advantage into account, we drop the absolute values in Eq. 6c. Cells operating in the upper right part of this plot have a competitive advantage for extracellular glucose, since these leave decreased residual glucose levels in the fermentor. Overall, we conclude that cells, being under limited substrate carbon conditions for a long time, increase the levels of those enzymes to compete for the available glucose in the environment.

**Figure 8:**
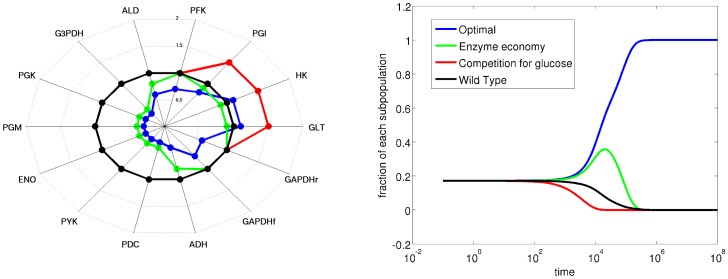
The competition experiment, to illustrate the optimal enzyme distribution considering the trade-off between enzyme economy and competitive ability for extracellular glucose. The radar plot on the left represents the enzyme levels, relative to wild-type, and the plot on the left represents the competition of each subpopulation with a specific enzyme setting as described in the radar plot. The color for each subpopulation is the same in both plots and is described in the legend.

To illustrate the advantage of considering the trade-off between enzyme economy and competitive ability, we designed a synthetic competition experiment. Four organisms differing by their enzyme levels are grown in a carbon limited chemostat, and the time course for each organism during this competition is simulated. The organisms are (1) wild-type, (2) only considering enzyme economy, (3) only considering competitive ability and (4) considering the trade-off between these two. The enzyme levels, relative to wild type and the time course of each organism are given in Figure 8. We find that the organism that considers the trade-off takes over the entire population in time.

Interestingly, we also predict that the evolved state is prompter for ATP response, i.e. in the evolved strain, ATP responds quicker to perturbations (Figure 7(D)). This is further confirmed by a glucose perturbation experiment (Figure 7(E), data taken from [41]). An important observation follows for PFK: based on both the function feasibility and the ATP temporal responsiveness feasibility, the level of PFK should decrease. However, if there is any decrease in the level of this enzyme, the cell can not survive in the chemostat, meaning that the cells are already at the edge of their feasible enzyme space for PFK (Figure 7(F)). Overall, the cells evolve to a state where they balance the competition for extracellular transport and getting rid of unused overcapacity. This transition makes the cells “specialists” in a specific condition, at the expense of loosing the capability of buffering large changes in the environment. We expect that FA will contribute to our understanding of trade-offs and the resulting evolutionary trajectories.

#### Feasibility analysis of alternative metabolic redesign in yeast glycolysis

To demonstrate how FA can serve to study metabolic (re)design, we consider the so-called turbo design in glycolysis. The turbo design is a general strategy followed by many catabolic pathways, consisting of first activating a substrate in a reaction that requires ATP, after which further metabolism yields a surplus of ATP [44]. In glycolysis, 2 ATP is initially invested in reactions catalysed by HK and PFK while 4 ATP are gained from reactions catalysed by PGK and PYK. The “danger” of this design is that when there is excess glucose, the upper part of glycolysis may run at a very fast rate that the lower part can not cope with. This can lead to accumulation of hexoses in the upper glycolysis (G6P, F6P, F16P), even though ATP and ADP are in steady state, resulting in substrate accelerated cell death [44].

**Figure 9:**
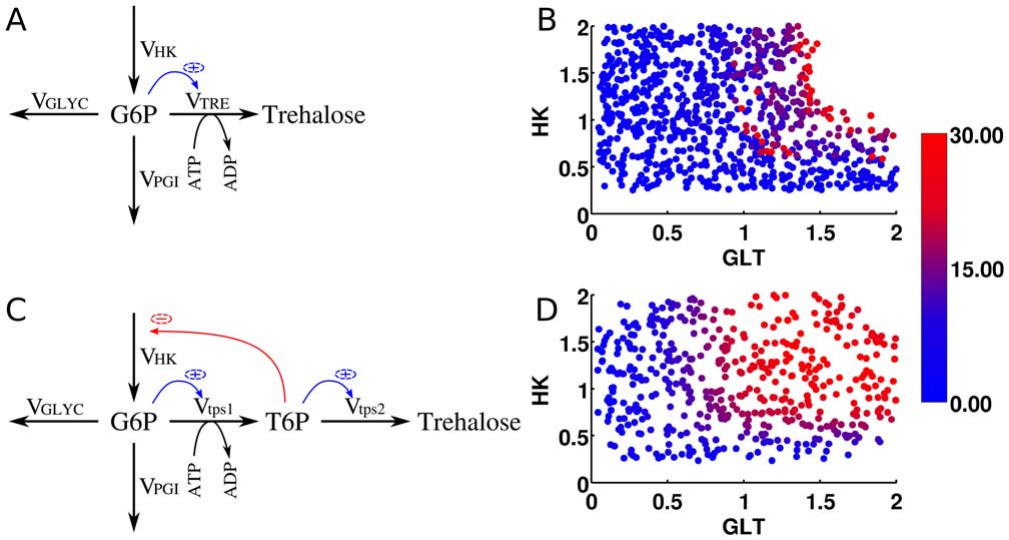
The danger of Turbo design and a potential solution investigated using FA. fig:TurboDesignAnalysisA: The original model considered in [24]. fig:TurboDesignAnalysisB: The feasible regulatory space of relative enzyme activities of HK and GLT. Increasing the enzyme levels leads to infeasible states for hexoses (red points on the upper right corner on the plot). fig:TurboDesignAnalysisD: The new design of the system with added metabolite T6P and its inhibition on HK. The new model parameters for storage branch are: 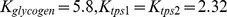. fig:TurboDesignAnalysisE: the same regulatory space as in fig:TurboDesignAnalysisB after addition of the feedback inhibition of T6P on HK. In fig:TurboDesignAnalysisA and fig:TurboDesignAnalysisD, only interactions within the focus are shown for simplicity where blue arrows indicate the kinetic activation and red arrow indicates inhibition. In fig:TurboDesignAnalysisB and fig:TurboDesignAnalysisE, only HK and GLT are monitored, remaining enzymes are held at their reference levels. The color code used in plots fig:TurboDesignAnalysisB and fig:TurboDesignAnalysisE is the co-response coefficient 
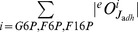
.

To illustrate the case, we consider the scenario where the cells are in glucose-rich conditions and inspect the homeostasis criterion of hexoses and ethanol flux (J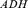). Figure 9(B) shows that in this initial design (Figure 9(A)) high levels of both GLT and HK (simulating a large load of substrate), are infeasible, as metabolite levels do not reach steady state. To resolve this handicap of the turbo design, we add a metabolite T6P and two reactions (tps1 and tps2) to the trehalose producing branch. We change the kinetic expression for HK, in line with [45], such that T6P inhibits HK via a feedback inhibition (Figure 9(C), see Methods for the new rate equation). The newly added reactions towards the trehalose pathway follow linear kinetics, and parameters are chosen to keep the metabolite and flux levels the same as the reference state (see caption, Figure 9). All other parameters remain the same as in [24]. A range of enzyme states that were previously infeasible become feasible with the new design (Figure 9(D)). When there is a large push of glucose, T6P acts as a “brake” to the glucose uptake, so that neither of the hexoses can increase uncontrollably.

Overall, our FA illustrates how a given metabolic design can be understood within the context of cellular objectives. An interesting observation on cellular trade-offs is that to overcome the danger of the turbo design, the cells have two options: increasing the capacity of reactions consuming the substrate (e.g. storage branches), or introducing T6P inhibition of HK. The first option is costly for the cell since the capacities of all enzymes in the storage pathway have to be increased. The second option is economical and homeostatically feasible, as already illustrated with the FA on the toy model. We finally speculate that evolution pushes cells to acquire this inhibition, in order to adapt to conditions where glucose levels significantly change. Note that such a “brake” system is not present for less-favorable carbon sources (e.g. maltose, [46]), an excess of which still results in substrate accelerated cell death.

Two main observations on FA follow from this illustration on the yeast glycolysis. First, for the prolonged chemostat scenario, although there is no prior fitting of model parameters to the experimental data, there is a remarkable quantitative correspondence between the enzyme data from long-term chemostat experiments and the prediction from FA using a dynamic model from literature. This clearly shows that FA can be used to explore the model, to evaluate alternative metabolic strategies, and to hypothesize about cellular trade-offs. Finally, mapping the experimental data clearly reveals which of the objectives is actually selected.

### Sampling based methods

We use a Monte Carlo sampling based method to construct the feasible enzyme space. Such a sampling based approach is, intuitive, unbiased and useful as illustrated by the examples. Similar methods are frequently used for exploring biological features, to build model families [18], [47], modeling of uncertainties in biochemical networks [19], robustness analysis [48], or designing synthetic networks [49]. Exploring the feasible space by sampling allows to study the trade-offs, and suboptimal behavior, frequently observed feature in biological systems [50]–[52].

Despite its advantages, sampling methods generally suffers from a number of limitations, mainly that they require lots of samples to cover the entire space, tend to waste too much effort and time on regions which are of no real interest and are therefore not scalable to large systems. In the future, our approach and the quantitative measures for feasibility can be combined with a smarter sequential sampling scheme, e.g. [53]–[55] to efficiently explore the initial space for a feasible sub-space.

### Feasibility analysis to study regulation at system level

Traditionally, regulation of metabolic networks is studied either by choosing a cellular objective for a genome scale model and optimizing the flux for that objective (top-down, FBA approach), or by constructing a kinetic model with detailed molecular interactions (bottom-up) and applying theorems of MCA or BST. Our method aims to combine elements of the two approaches and allows to study objectives other than flux. Furthermore, as we propose quantitative objectives for feasibility, rather than studying “viable” or “lethal” changes, we can study sub-optimality and trade-offs. By exploring the feasible enzyme space, FA allows evaluating alternative hypotheses and interpreting experimental data.

FA assumes the availability of a kinetic model. Despite a long list of challenges, e.g. a high degree of non-linearity, lack of sufficient experimental data, coexistence of multiple time scales etc. [16], [56], currently available information on the kinetics of individual enzymes [57] as well as the list of available reliable kinetic models is increasing [58]–[60]. Our example with the detailed kinetic model of glycolysis illustrates how FA can be applied to realistic problems. This is urgently needed, given the growing accumulation of experimental data obtained from different omics layers of cells. Tools to analyze such data are of high value.

Previous computational efforts to understand regulation of metabolism include searching for design principles using optimization principles [23], exhaustively searching and identifying enzyme-based motifs while seeking adaptive properties in a library of network topologies [61], designing synthetic networks for specific tasks [49] or the use of constraints in kinetic parameters to constrain the solution space in steady state models [62]. In particular, the approach taken by Sorribas and co-workers is similar to our FA approach, in that they also investigate feasible enzyme activity patterns leading to cellular (adaptive) responses [63]. Their analysis efficiently finds a global optimum for a given objective, under a given list of physiological constraints. Their mathematically involved approach is tightly coupled to the GMA formulation, elegantly exploits the mathematical structure of the non-convexities of the model. This coupling, in turn, limits its general use. Our proposed FA differs from [63] and [23] in two points. First, it is more general and can be used with any system that can be simulated with a model. Second, central to FA, we propose and use generic quantitative measures for cellular objectives, aiming to eliminate *ad hoc* definitions. This allows us to consider objectives other than thresholds on fluxes or concentrations, such as robustness/homeostasis and temporal responsiveness.

### Conclusions

In this paper, we addressed the following question: being under constraints and evolutionary pressure, why and how is metabolism regulated? To answer this question, we took a top-down approach and speculated that cells, under physiological constraints, are regulated to optimize (one or a combination of) a number of objectives and can hence only assume enzyme levels falling in a so-called feasible enzyme space. We further analyzed how metabolism should be designed from a feasibility perspective, i.e. we addressed the question what are the necessary kinetic interactions in order for cells to attain an objective. Unique to our approach, we proposed quantitative metrics to measure proposed cellular objectives.

One of the fundamental characteristics of biological systems, homeostasis, requires globally coordinated regulation of enzyme levels. An interesting observation for homeostatically feasible states is that these fall in two distinct sub-regimes: a low-flux regime, where all enzymes are downregulated, and are less costly for the cell; and a high-flux regime, where all enzymes are upregulated, therefore costly for the cell. The actual regime chosen by the cell is defined with respect to the environment. In the prolonged chemostat scenario, the cell optimizes the enzyme levels for function, since the carbon influx is externally kept constant. From an enzyme budget point of view, the ubiquitously present feedback inhibition is an economical way to ensure homeostasis. This is especially important for keeping metabolite levels within limits upon a wide range of fluctuations in the environment.

In contrast to homeostasis, maintaining robustness requires a local metabolic effect, meaning that the function can still be maintained by locally adjusting the metabolite levels around specific perturbed enzymes. In line with our findings, Sauer and co-workers recently showed in yeast that alterations in enzyme capacity are buffered by converse changes in substrate metabolite concentration, thereby minimizing the difference in metabolic flux caused by the alteration [52]. In this work, we took homeostasis or robustness as objectives so that we could also study sub-optimal states and the trade-offs between various objectives. This is in contrast to previous attempts where homeostasis has been considered as constraint for the metabolic design problem [15].

Temporal responsiveness reflects a dynamic property of the system. We speculate that this objective is especially applicable to networks whose dynamic properties are of evolutionary importance, e.g. ultrasensitivity, response time etc. As an example, for signaling pathways the effect of network structure on dynamic properties has already been discussed [37], [38], [64]. Note, that our approach can equally well be used for any other kinetic model, although the physiological objectives may need to be customized. The objective functions we have formulated in this study are illustrations of a more general approach: it may as well be that other objectives turn out to be more relevant under different conditions. It should also be noted that, here, we proposed three “container” objectives that are physiologically relevant, which need to be further specified depending on the case evaluated. Additional quantifiable objectives such as overcapacity (which may be defined as the ratio of actual flux to the maximum possible flux) can easily be considered as well.

Taken together, we see that FA quantitatively evaluates alternative hypotheses, shows trade-offs between the available objectives and provides an intuitive platform to integrate the proteome information (enzyme space) with information on metabolome and fluxome (physiological space). Such an integrative approach is indispensable to analyse and interpret the increasingly available multi-omics data on regulation of metabolic networks especially when considering optimal performance or adaptation in response to external stimuli. We illustrated quantitatively via FA that there is a very limited set of enzyme set that are feasible for all the considered objectives. Similar to [51], we argued that the cells are often faced with trade-offs between alternative strategies. Furthermore, by fully exploring the initial viable space and quantitatively evaluating physiological objectives, we got insights on how the metabolic systems are designed (e.g. the “brake” for temporal responsiveness objective). This aspect is similar to the “design space for biochemical systems” concept in [65], [66], but has the additional benefit of direct use of the physiological objectives, making the link from genotype to phenotype more intuitive.

## Methods

### Feasible enzyme space

To construct the feasible enzyme space, we first quantitatively formulate the constraints and the objectives for physiological states. Then we calculate the range of *theoretically possible* physiological states, and call this the viable enzyme space. We then select a *feasible* subspace based on the pre-defined criteria and analyze the properties of this subspace. Overall, we construct the feasible enzyme space 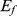 for enzyme levels 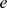 as
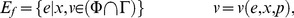
(3)where 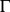 is the set of viable states considering the constraints, 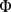 represents the set of feasible physiological states (metabolites and fluxes (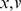)) considering the list of physiologically relevant cellular objectives, and 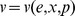 is the rate of the reaction catalyzed by the enzyme 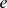 as a function of the enzyme level 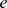, metabolite level 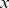 and set of parameters 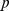. The constraints and objectives are detailed below. Some of the metrics for these constraints and objectives are defined with respect to a so-called “reference state”, denoted with superscript “^0^”. This way, all defined entities can be measured with respect to this reference state, much like the elasticity parameters or control coefficients in MCA literature. Conventionally, the reference state can be chosen as the steady state that cells achieve when they are grown under constant, substrate limited conditions and is usually characterized by intracellular fluxes, metabolites and enzyme levels.

The feasible enzyme space constructed is, in fact, a sampling of a multidimensional space containing enzymes, metabolites and fluxes. We visualize this space by slices, i.e. 2D cross-sections. A GUI written in Matlab, and supporting functions as well as the datasets mentioned in this paper can be downloaded at: http://bioinformatics.tudelft.nl/. The interface takes as input a dataset, calculates the feasibility criteria for selected objectives of the cell and visualizes by (selected) slices.

#### Constraints: Thermodynamic constraints

Thermodynamic constraints are formulated as irreversibility constraints for fluxes through reactions operating away from equilibrium:
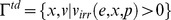
(4a)When more quantitative information is available, for example when Gibbs free energy of a reaction is known, this can also be taken into account as
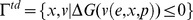
(4b)Note, that in a kinetic model in which the equilibrium constant is incorporated in the rate law (e.g. implicitly through the Haldane-relationship), this constraint would already be taken into account.

#### Constraint on total enzyme level

We constrain the total enzyme level to change in a limited range, while individual enzymes can freely be interconverted:
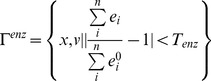
(5)where 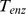 is a precision parameter (e.g. 0.1), and 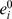 is the enzyme level for reaction 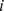 at a reference state (denoted by “^0^”). This criterion demands that the total enzyme level stays nearly constant (e.g. can change only within 10%, when 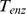 = 0.1).

#### Cellular objectives: Function

We define states in which near-optimal flux under constrained enzyme levels is obtained as feasible:
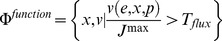
(6a)where 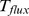 is a cut-off value for feasibility in terms of optimal flux. This criterion demands that a flux can be at most 10% (when 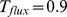) away of it's possible maximal flux (denoted as 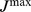).

#### Robustness and Homeostasis

We consider *robustness* as a property that allows a system to maintain its function under perturbations and *homeostasis* as the coordinated physiological processes which maintain the current state [28]. In this work, the perturbations are changes in enzyme levels, states are metabolite levels and function is the flux towards a selected pathway or reaction. In order to quantify both robustness or homeostasis, we need a measure between state (metabolite levels), function (flux towards the selected enzyme/pathway) and perturbation (changes in enzyme levels). We use the co-response coefficients (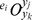) as a measure, defined within the context of Metabolic Control Analysis (MCA) [67] as:
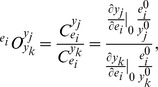
where 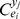 is the control coefficient of feature 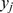, defined as the scaled sensitivity coefficient of 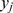 towards the enzyme 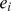. The co-response coefficient describes the effect of a perturbation in enzyme 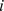 on both features 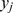 and 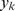. For example, 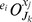 denotes the co-response coefficient of metabolite 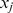 and flux 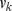 upon changes in enzyme 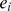. Note that metabolite 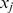 and reaction rate 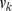 need not be connected by a kinetic expression; the co-response coefficient describes a *network* property, rather than a local property such as the elasticity of a reaction towards a substrate or product.

Focusing on *robustness*, the definition implies that the effect of the perturbed enzyme on the target flux (the function) should be small, i.e. 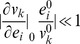. In this case an enzyme perturbation would have an effect on the metabolite levels only, i.e.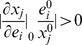. The resulting co-response coefficient should therefore be large:
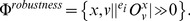
(6b)Second, *homeostasis* is considered. A state is called feasible if upon enzyme perturbation, metabolite levels do not change significantly 
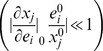
, whereas the flux does. We formulate the feasibility related to homeostasis for a set of 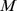 metabolites as:
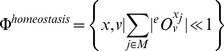
(6c)The summation over the metabolites ensures that homeostasis is not only local in one metabolite but over a number of relevant metabolites, e.g. belonging to a pathway.

#### Temporal responsiveness

Temporal responsiveness of metabolite levels in a metabolic network in response to perturbations is defined using the turn-over time of metabolites
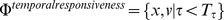
(6d)with 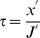, 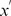 and 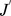 the physiological parameters at the steady state reached after a perturbation and 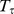 is a treshold. This criterion demands that the turnover time of a metabolite should be smaller than e.g. 0.5 per unit time, when 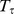 = 0.5.

### Regulation analysis

An important question is to what extent metabolic fluxes are regulated by gene expression or by metabolic regulation. In line with the convention in regulation analysis [4], “metabolic” regulation is defined as change caused by concentrations of substrate(s), product(s) and modifier(s). “Hierarchical” changes are those caused by change in enzyme concentration, via alterations in mRNA sequestration and intracellular localization and/or rates of transcription, translation or degradation. Both types of regulation are quantified by the hierarchical and metabolic regulation coefficients (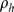 and 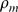) defined as
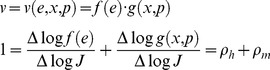
(7)


We consider cases where 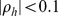 as exclusively metabolically regulated, and cases where 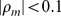 as exclusively hierarchically regulated. One immediate application of information from regulation analysis is in metabolic engineering. In the first case, increasing the flux could be achieved by simply increasing the enzyme level whereas for the second case, alternative engineering strategies such as protein engineering to change the kinetic properties of the enzyme need to be considered.

### Illustrative cases

#### Toy model

To illustrate feasibility analysis, we use a small example model with tractable kinetics, where a substrate 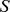 is converted into a product 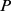 via a linear pathway of 3 reactions and 2 intracellular metabolites (Figure 2). The model assumes a steady state and all three rates follow linlog kinetics, allowing to calculate an explicit steady state solution for metabolites and rates in terms of enzyme levels and kinetic parameters [12]. In linlog kinetics, the rate of reaction 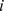 (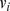) is described relative to the steady state flux 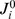 as a function of the enzyme levels 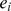 and intracellular and extracellular metabolites (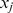 and 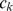) all relative to their steady state levels 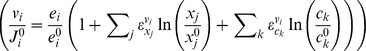. The reference steady state conditions (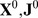) and the elasticity matrix 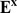 composed of kinetic parameters (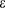) are given in Figure 2.

#### Glycolysis model in yeast

To study feasibility analysis applied on a real problem, we used a previously published model of glycolysis in *Saccharomyces cerevisiae* [24]. The kinetic expressions for each reaction and the parameters are the same as [24], and the reference state used in our work is given in Table 1. For feasibility analysis, we need to sample the enzyme levels, relative to their reference state and in the yeast model, this is performed by sampling the relative V
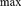
'es since:
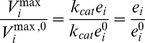
where, superscript ^0^ is the corresponding entity at the reference state.

**Table 1:** The reference conditions for the yeast problem.

Fermentation parameters	
D = 0.05 hr^−1^, Glucose 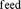 = 210 mM Biomass = 15 gDW^−1^	
Intracellular independent fluxes (mmol L 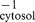 min^−1^)	
vglt	0.185
vglyc	0.011
vtr	0.00431
vatp	0.06335
Intracellular metabolite concentrations (mmol L 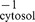 )	
GLCi	0.032508
P	0.65319
G6P	0.048434
F6P	0.0092476
F16P	0.0046687
TRIO	0.029825
NADH	0.17489
BPG	1.68 10^−6^
P3G	0.0071666
P2G	0.00084789
PEP	0.0014801
PYR	0.72143
ACE	0.028478

The reference conditions for the yeast problem.

For the feasibility analysis of alternative metabolic redesign in yeast glycolysis, the new kinetic expression for the HK reaction is:
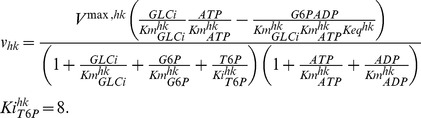



Lastly, in order to illustrate the competition in the fermentor, we added a growth equation for this and expressed the growth rate with simple monod-growth kinetics as:
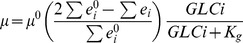
with 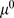 is the growth rate at the reference conditions (and is equal to the dilution rate in the chemostat) and 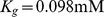, the extracellular glucose level at the reference conditions. The term between the parantheses represent the effect of total enzyme cost on growth.

### Data pre-processing

In using experimental data, there were multiple measurements for a specific time point. Since the data available was insufficient to assume and fit a parametric model, we used non-parametric Gaussian kernel regression 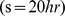 to estimate the average at a specific point, taking all data into account.
